# La trayectoria de vida de Irma Carrica: una aproximación a los debates político-sanitarios de las décadas de 1960 y 1970 desde el método biográfico

**DOI:** 10.18294/sc.2024.4779

**Published:** 2024-06-25

**Authors:** Mariano Gabriel Vigo Deandreis, Manuel Fonseca

**Affiliations:** 1 Profesor de Enseñanza Media y Superior en Historia. Ayudante de Primera, Departamento de Ciencias Económicas y Jurídicas, Universidad Nacional de Moreno. Buenos Aires, Argentina. mvigodeandreis@gmail.com Universidad Nacional de Moreno Departamento de Ciencias Económicas y Jurídicas Universidad Nacional de Moreno Buenos Aires Argentina mvigodeandreis@gmail.com; 2 Médico especialista en Medicina General y Familiar. Docente, Facultad de Ciencias Médicas, Universidad Nacional de La Plata. Prosecretario de Salud de la Presidencia, Universidad Nacional de La Plata. Maestrando, Maestría en Epidemiología, Gestión y Políticas de Salud, Instituto de Salud Colectiva, Universidad Nacional de Lanús, Buenos Aires, Argentina. mfonseca@med.unlp.edu.ar Universidad Nacional de La Plata Facultad de Ciencias Médicas Universidad Nacional de La Plata Buenos Aires Argentina mfonseca@med.unlp.edu.ar; Instituto de Salud Colectiva Universidad Nacional de Lanús

**Keywords:** Historia, Salud Comunitaria, Biografía, Activismo Político, Argentina, History, Community Health, Biography, Political Activism, Argentina

## Abstract

Reconstruimos la trayectoria de vida de la enfermera y militante popular argentina Irma Carrica, entendida como una experiencia político-profesional ligada a sus redes de sociabilidad y atravesada por conflictos y contradicciones inherentes a su contexto histórico. Desde ese recorte analítico y teniendo en cuenta los recaudos sugeridos por el método biográfico de las ciencias sociales, nos adentramos en los debates político-sanitarios de las décadas de 1960 y 1970, principalmente en lo relativo a las disputas por el sentido de la “comunidad” en el campo de la salud. En particular, nos enfocamos en las contribuciones de un actor histórico colectivo -heterogéneo y plural, pero identificable en sus distintas modulaciones- que hemos denominado izquierda peronista en salud. Al analizar sus redes profesionales e intelectuales, hicimos hincapié en el rol que desempeñó Irma Carrica como referente de esa izquierda peronista en salud, a la hora de construir dinámicas alternativas para el abordaje comunitario en salud, que pusieron en tela de juicio los paradigmas epistemológicos y pedagógicos dominantes.

## Abstract (en)

We reconstruct the life path of the Argentine nurse and popular activist Irma Carrica, understood as a political-professional experience tied to her social networks and marked by conflicts and contradictions inherent to her historical context. From this analytical perspective and considering the precautions suggested by the biographical method of social sciences, we delve into the political and health debates of the 1960s and 1970s, particularly concerning disputes over the meaning of “community” in the health field. Specifically, we focus on the contributions of a collective historical actor - heterogeneous and plural, yet identifiable in its various forms - that we have termed the Peronist Left in health. By analyzing their professional and intellectual networks, we emphasize the role played by Irma Carrica as a representative of this Peronist Left in health, in constructing alternative dynamics for community health approaches, which challenged the dominant epistemological and pedagogical paradigms.

## INTRODUCCIÓN

En el presente artículo reconstruimos la trayectoria política, sindical y profesional de Irma Laciar de Carrica, como una puerta de entrada a los conflictos y debates político-sanitarios latinoamericanos de las décadas de 1960 y 1970. En particular, nos preocupamos por abordar los clivajes que vertebraron las disputas por el sentido de “la comunidad” en el campo de la salud, haciendo hincapié en los aportes realizados desde los núcleos de sociabilidad político- profesionales que integró Irma Carrica y que pretendían abonar un proyecto alternativo de organización y agenciamiento comunitario en torno a las problemáticas de salud.

La emergencia de esos debates se dio en un contexto particular, atravesado por cuestiones económicas y geopolíticas mutuamente imbricadas. En materia económica, entre mediados de la década de 1950 y la primera mitad de la década de 1960, empezó a consolidarse la hegemonía de un paradigma desarrollista de gestión estatal, en desmedro del llamado Estado de bienestar. De tal suerte, si en el pasado la vocación de centralizar la gestión sanitaria se había apoyado en un modelo universalista de salud pública, ahora el Estado debía dotarse de cuadros técnicos que colaboraran con un proceso de descentralización del sistema y “racionalización” del uso de los recursos públicos^1^.

En el caso argentino, el Estado de bienestar tuvo lugar durante las dos primeras presidencias de Juan D. Perón (1946-1955) y, en materia de salud, con la gestión del primer ministro de salud de la Argentina, el doctor Ramón Carrillo. A raíz de su intervención y bajo el influjo universalista de la “seguridad social” británica, el Estado asumió un rol preponderante como garante del acceso igualitario y gratuito a la salud. De tal suerte que, por primera vez en la historia de nuestro país, hubo una política integral e integrada de salud que, a pesar de entrar en contradicción con el peso y la lógica del seguro social sindical, contó con un desarrollo infraestructural y técnico acorde a los desafíos del periodo^2^.

Al abordar esta etapa, veremos cómo Irma Carrica, desde sus espacios político-profesionales y sindicales de pertenencia, fue desarrollando una concepción particular sobre la salud pública y las lógicas de intervención sanitaria, aún como opositora al peronismo. 

Años después, en consonancia con los intereses locales y extranjeros que impulsaban la transición hacia un paradigma desarrollista, el golpe de Estado perpetrado por la autoproclamada “Revolución Libertadora” (1955-1958) ensayó una revisión del periodo anterior, imbuida por el afán de desterrar el influjo del justicialismo. Bajo este prisma, impulsó una serie de medidas para garantizar la proscripción política del peronismo y anular sus distintas expresiones organizativas y simbólicas. A su vez, en materia de salud, eliminó gran parte del acervo material y organizativo legado por la gestión de Carrillo^1^.

Como contraparte, todas estas medidas dictatoriales dieron lugar a una respuesta de raigambre popular y relativamente espontánea, que dio origen a la llamada Resistencia Peronista (1955- 1973). Teniendo en cuenta ese contexto, analizaremos las experiencias profesionales, políticas y sindicales que acompañaron y modularon el proceso de peronización de Irma Carrica y su rol durante la primera etapa de la Resistencia (1955-1966).

Por otro lado, en lo relativo al contexto geopolítico, la declaración del carácter socialista de la Revolución Cubana de 1961 exacerbó la traslación de la lógica bipolar de posguerra hacia nuestro continente. De modo que, frente al potencial avance del comunismo en la región, los organismos internacionales que estaban bajo la órbita de EEUU promovieron distintas iniciativas de contención y disciplinamiento social. Entre ellas, la más significativa fue la llamada “Alianza para el Progreso” de 1961.

La vigencia de esta clase de enfoques tuvo un correlato significativo con las reformas internacionales que bregaban por correr el eje del debate hacia una variante restrictiva de salud comunitaria. A los fines de nuestra investigación, es de particular importancia la idea de “descentralización comunitaria” impulsada por la OMS/OPS y apoyada en las directrices del Concilio Vaticano II. En Argentina, esa idea se plasmó en la Ley 17102 de 1966, en un marco de continuidad de la proscripción del peronismo y bajo el gobierno de facto de Juan Carlos Onganía. Esta ley hacía hincapié en la necesidad de que la sociedad civil se hiciese cargo de canalizar la resolución de los problemas en materia de salud. En consecuencia, el Estado debía conformar equipos técnicos interdisciplinarios de asesoramiento y control, con el objetivo de delegar la gestión sanitaria en las comunidades o en la iniciativa privada^2^. De tal suerte, la participación de la población funcionaría como fuente de información y medio de aplicación de políticas guiadas por criterios técnicos ajenos a la voluntad popular^1^.

Mientras las corrientes de salud comunitaria que buscaban sostener el *statu quo* ponían el énfasis en lo técnico-profesional y en la enseñanza interdisciplinaria y extrahospitalaria, las perspectivas alternativas hacían hincapié en estimular la participación social como piedra angular para repensar el sistema de salud en su conjunto^3^.

Para el caso argentino, estas disputas tuvieron lugar entre 1966 y 1974. Durante ese periodo, las distintas redes político-intelectuales y profesionales que integró Irma Carrica formaban parte de lo que en trabajos anteriores hemos caracterizado como “izquierda peronista”^4^^,^^5^ y que, en razón de nuestro recorte, podríamos denominar izquierda peronista en salud.

Para adentrarnos en los debates aludidos y en las prácticas político-sanitarias alternativas que prohijaron, elegimos recuperar la trayectoria de vida de Irma Laciar de Carrica, entendida como una experiencia político-profesional ligada a sus redes de sociabilidad y atravesada por conflictos y contradicciones inherentes a su contexto histórico. Bajo este prisma, los interrogantes que guían nuestra pesquisa podrían resumirse del siguiente modo: ¿Qué posturas adoptó Irma Carrica, desde sus núcleos de sociabilidad político-profesional, en las distintas etapas de la disputa por el sentido de “la comunidad” en el campo de la salud?; ¿con qué otros actores antagonizó?; ¿qué rol le asignaba al Estado en el abordaje de las problemáticas de salud?

## CONSIDERACIONES METODOLÓGICAS

Para abordar nuestro interrogante, trabajamos con un total de cinco entrevistas semiestructuradas realizadas a actores claves del periodo, contrastando sus testimonios con otras fuentes complementarias como revistas, entrevistas fílmicas, registros oficiales, documentos familiares y de agrupaciones políticas.

Una de las entrevistas fue realizada por María Lucrecia “Maluca” Ciriani, enfermera militante de la izquierda peronista en salud, el 31 de marzo del 2001, a Héctor “Pelusa” Carrica, hijo de Irma Carrica. Otra de las entrevistas a Héctor “Pelusa” Carrica fue realizada en octubre de 2018 por la licenciada Florencia Paparone, en el marco de otro proyecto. Las entrevistas restantes fueron realizadas entre marzo y abril de 2023: el 7 de marzo a María Lucrecia “Maluca” Ciriani, enfermera militante de la izquierda peronista en salud (duración aprox.: 86 minutos); el 13 de marzo a Marcela “Hormiga” Durrieu, cofundadora de la Juventud Universitaria Peronista (JUP) en la Facultad de Medicina de la Universidad de Buenos Aires (duración aprox.: 67 minutos); el 5 de abril a Ernesto Villanueva, referente intelectual de la Izquierda Peronista y vicerrector de la entonces denominada Universidad Nacional y Popular de Buenos Aires (UNPBA) (duración aprox.: 50 minutos). A dichas entrevistas, se agregan las conversaciones informales con: Graciela Ríos, esposa de Héctor “Pelusa” Carrica; Noemí Viveros, integrante del grupo “Docentes Grierson por la Memoria”; y la licenciada Beatriz Morrone, especialista en historia de la enfermería argentina.

Todas las entrevistas fueron grabadas, ya sea con dispositivos celulares o a través de videollamadas por computadora. Las grabaciones fueron transcriptas por los autores sin utilizar programas de transcripción automática. Las personas entrevistadas fueron notificadas del objetivo de las entrevistas y dieron su consentimiento para su utilización en el presente artículo.

Al analizar estas fuentes testimoniales, partimos de los postulados de Pierre Bourdieu sobre los vínculos entre (auto)biografía, memoria y ciencias sociales. Según este autor, tanto el investigador como el testimoniante están compelidos por una tentación narrativa que los conduce hacia una “ilusión biográfica”. En ella, los acontecimientos se encadenan de manera lógica y necesaria, de modo que la vida aparece como una existencia dotada de sentido, con un principio (origen y razón de ser, a la vez) y un fin (desarrollo necesario y meta a alcanzar, simultáneamente). Para eludir esa tentación, el autor propone la noción de “trayectoria de vida”, entendida como una serie de posiciones sucesivas ocupadas por un mismo agente dentro de un espacio social en devenir, atravesado por conflictos, contradicciones e imprevistos^6^.

En ese sentido, nuestro abordaje sobre la trayectoria de vida de Irma Carrica reconoce tres niveles de análisis: la existencia de un “yo” (nivel microsocial), que influye en y se ve afectado por relaciones intersubjetivas en el marco de grupos e instituciones (nivel mesosocial), cuyas dinámicas están atravesadas por contextos históricos específicos (nivel macrosocial). Bajo este prisma, nos concentramos en el vínculo histórico entre los niveles micro y mesosocial de análisis, siguiendo dos estilos de investigación propios del “método biográfico”, a saber: el de la *microhistoria*, que pone en valor la recuperación de memorias no hegemónicas para reconstruir procesos colectivos de gran significación histórica; y el de las *marcas narrativas*, cuya finalidad es la de indagar en las tensiones creativas que anudan las dimensiones sincrónica y diacrónica del relato autobiográfico en la construcción identitaria del testimoniante^7^.

A la hora de analizar las *marcas narrativas* de los testimonios, tomamos en cuenta los recaudos sugeridos por Leonor Arfuch, quien señala que la “identidad narrativa” del yo testimonial se construye a partir de una articulación fluctuante entre lo individual y lo colectivo, entre la parcialidad del punto de vista y el contrato de veridicción del testigo. La situación es tanto más compleja en la medida que hablamos de la elaboración discursiva de experiencias atravesadas por el influjo traumático del golpe de Estado de 1976 en Argentina. En cualquier caso, el “yo narrativo” es el mismo y otro cada vez que se narra, por lo que -más allá de su creencia- no cuenta siempre la misma historia^8^. Muy por el contrario, en el discurso que anuda “atención y memoria activa” con “repetición y memoria pasiva”, se produce la intelección de la propia historia, es decir: la reactualización de la relación entre pasado, presente y futuro^9^.

Asimismo, al analizar el devenir histórico de las redes político-intelectuales y profesionales de las que Carrica formó parte, en el marco de la izquierda peronista en salud, tomamos en cuenta otra serie de recaudos. Por un lado, es importante señalar que los vínculos de pertenencia podían ser múltiples y no necesariamente excluyentes, dado que los equipos político-técnicos eran unidades abiertas en relación con un problema. De modo que, más allá de compartir ciertas cosmovisiones en común, su fortaleza residía en la posibilidad de armarse y desarmarse haciendo cosas en torno a un conflicto y sus aristas^10^^,^^11^.

Bajo este prisma, hemos identificado tres etapas en el desarrollo del proyecto sanitario de la izquierda peronista en salud. Durante la primera fase, entre 1966 y 1971, se manifestaron distintas experiencias vinculadas a la disputa por el sentido de “la comunidad” en el campo de la salud, que confrontaban con el espíritu de las iniciativas de “descentralización comunitaria” y le dieron forma a una incipiente izquierda peronista en salud. En un segundo periodo, de 1971 a 1973, esa izquierda peronista en salud fue elaborando una sistematización teórica de las experiencias de la etapa anterior, principalmente en el marco de los debates que tuvieron lugar en la revista *Ciencia Nueva*^12^. Finalmente, entre 1973 y 1974, se dio una cristalización parcial del proyecto de la izquierda peronista en salud, en el marco de la gestión de la Universidad Nacional y Popular de Buenos Aires (UNPBA)^13^ y del Ministerio de Salud y Acción Social de la Provincia de Buenos Aires^14^. En todas estas etapas, como veremos, Carrica desempeñó un rol de articulación fundamental entre las esferas político-intelectuales y profesionales más o menos institucionalizadas de la izquierda peronista en salud y las organizaciones territoriales de militancia.

## IRMA CARRICA

### Los pasos previos en su fragua identitaria (1945- 1966)

Irma Carrica nació el 3 de julio de 1926 en Sampacho, provincia de Córdoba. Su madre, Asunta Pericas, murió muy joven, quedando ella y su hermano menor bajo la tutela de su padre, Máximo Laciar. Él trabajaba como ferroviario en Mercedes (San Luis), donde fue elegido delegado del gremio. Mientras cursaba sus estudios primarios en Córdoba, Carrica creció -según el testimonio de su hijo- bajo el influjo de sus tías maternas, ambas enfermeras. Con la impronta de esa crianza, migró a Buenos Aires. Fue allí donde completó el secundario y decidió adentrarse en el campo de la salud. Según el mismo testimonio familiar, aspiraba a convertirse en médica, cosa que no pudo conseguir porque tuvo un ataque de *surmenage* que le impidió afrontar tal exigencia intelectual.

No obstante, en el año 1947, se recibió de enfermera en la Escuela “Cecilia Grierson”. Este dato generó ciertas suspicacias entre algunas entrevistadas, producto de la reticencia de las autoridades institucionales a habilitar el acceso al archivo de Carrica (Noemí Viveros, docente de la Escuela “Cecilia Grierson”; María Lucrecia “Maluca” Ciriani, enfermera militante de la izquierda peronista en salud). Sin embargo, luego de consultar su “legajo reparado”^15^, constatamos que cursó como “alumna becada” entre 1945 y 1947. Una vez recibida, regresó a Córdoba y comenzó a trabajar en el Hospital Central de Río Cuarto. En ese tiempo conoció a Héctor Luis Carrica, con quien se casó en diciembre de 1947. A su vez, en septiembre de 1948, tuvieron a su primer y único hijo, Héctor “Pelusa” Carrica.

Cuatro años más tarde, cuenta “Pelusa”, Carrica se separó y regresó a Buenos Aires. Al tomar esa decisión, se vio en la necesidad de dejar a su hijo en un colegio pupilo. Después de un tiempo de trabajar en Buenos Aires, pudo traerlo y lo crio sola, entre el trabajo y el estudio. De tal suerte, se fue afianzando un vínculo muy fuerte entre ellos, cimentado en las experiencias vividas.

Para ese entonces, como hemos señalado, se consolidó un enfoque alternativo de salud pública, ligado a la gestión de Ramón Carrillo, que fue acompañada por un estímulo estatal para la profesionalización de la enfermería, cuya importancia venía creciendo desde 1940. Así, luego de sancionado el Decreto 6216 de 1945, que reglamentó el ejercicio de la medicina y sus “ramas auxiliares”, la entonces Secretaría de Salud Pública, en 1947, impulsó la creación de una Escuela de Enfermería, con la intención de “...formar personal auxiliar de la medicina, técnica y moralmente capacitado”^16^. Asimismo, en 1948, Eva Perón creó la Escuela de Enfermería de su Fundación, con un perfil que combinaba la capacitación técnico-profesional con la formación política. Y si bien suele argüirse que los perfiles de las instituciones aludidas antagonizaban en torno a un clivaje que enfrentaba lo técnico con lo político, ambas contaban con planes de estudio que hacían hincapié en la perspectiva de la salud pública, la formación extrahospitalaria y el enfoque social de la práctica sanitaria, que no tenían antecedentes en nuestro país^16^.

Para ese entonces, en 1953, Carrica entró a trabajar en el Hospital General de Agudos Bernardino Rivadavia, donde continuó formándose hasta llegar a ser enfermera principal y subjefa de enfermería, pasando primero por el rol de instrumentadora. Asimismo, como opositora al peronismo y habiendo militado desde joven en el Partido Socialista, liderado por Alfredo Palacios, se afilió al sindicato Asociación de Trabajadores del Estado (ATE) y llegó a ser secretaria general del gremio en el Hospital^17^.

En ese contexto, según el testimonio de “Pelusa”^18^, Carrica habría sido convocada para dictar cursos en la Escuela de Enfermería de Carrillo. Sin embargo, conforme el análisis de sus legajos institucionales^15^^,^^19^, no hemos podido constatar tal participación. A su vez, producto de la lógica de selección que -bajo la dirección de Teresa Molina- primaba en dicha Escuela, resultaría poco probable que hayan aceptado la participación de una militante socialista y madre soltera^17^. No obstante, sí observamos en Carrica una temprana preocupación por garantizar el acceso de los sectores populares a la salud pública, junto con un interés por la docencia compatible con dicha preocupación.

Por otro lado, el ya mencionado proceso de profesionalización de la enfermería estuvo atravesado por dos contradicciones: la feminización y precarización laboral de la profesión, y su dilución dentro de la hegemonía médica. Al naturalizar la feminización de estas labores y su estatus complementario respecto del salario masculino, las mujeres pobres se vieron compelidas a tomar puestos mal remunerados y precarios en enfermería, a la espera de ganar mayor reconocimiento y estabilidad.

Frente a ese escenario, según “Pelusa”, una de las luchas sindicales impulsadas por Carrica fue la de promover la formación del personal empírico en horario de servicio, junto con el reconocimiento de su real función. Si bien esto se dio en un contexto más favorable que el que vendría luego, los distintos esfuerzos por jerarquizar el oficio -tanto sindicales como corporativos- chocaron con las matrices ideológicas que alimentaban el imaginario sobre la enfermería. En esa dicotomía, los primeros intentos de profesionalización fracasaron, aunque sentaron las bases para futuras conquistas y procesos organizativos de lucha.

### Transición hacia el desarrollismo y primera etapa de la resistencia (1955-1960)

En las postrimerías del segundo mandato de Perón, la recuperación económica que se había obtenido con el Plan de Estabilización contrastaba con el deterioro de la situación política. Por un lado, la convocatoria a una convención constituyente para establecer la separación entre la Iglesia y el Estado -junto con la derogación de la ley de enseñanza religiosa y moral en las escuelas públicas- exacerbó el encono que las autoridades eclesiales arrastraban con el gobierno. A su vez, el impacto de las negociaciones petroleras le hizo perder apoyos nacionalistas dentro de las fuerzas armadas. La convergencia de estos dos actores institucionales habilitó una nueva conspiración cívico-militar, a la que se sumaron el resto de las fuerzas de oposición preexistentes^20^.

Dentro de las fuerzas armadas, la principal impulsora del intento de golpe de Estado fue la Armada Argentina, cuyo objetivo era asesinar a Perón. Al no contar con el apoyo mayoritario del Ejército y la Fuerza Aérea, la iniciativa se frustró. No obstante, el día del intento - el 16 de junio de 1955- las bombas que arrojaron sobre la sede gubernamental y el Ministerio de Guerra dejaron un saldo de mil víctimas, entre muertos y heridos, la mayoría de ellos, civiles^20^.

En distintas entrevistas, “Pelusa” señaló que este acontecimiento marcó a fuego la trayectoria posterior de su madre, dado que -como enfermera del Hospital Rivadavia- le tocó socorrer a las víctimas del bombardeo. En palabras de “Pelusa”:

“*Un relato de ella que me quedó grabado* [...] *era cuando nos contaba que el cuadro en la Plaza de Mayo era dantesco. Había un transporte escolar* [...] *y a la maestra que llevaba el contingente le explotó una bomba, perdiendo así las piernas* [...] *y gritaba: ‘¡saquen a los chicos!’* [...] *pero los chicos estaban muertos*”. (Héctor “Pelusa” Carrica, marzo 2001)

En ese escenario, se encontró con la desidia y la complicidad de los médicos que convalidaban el golpe de Estado, situación que contrastaba con el sufrimiento del pueblo con el que empatizaba y del que se sentía parte. Siguiendo esta perspectiva, “Pelusa” señaló que:

“*Otra vivencia que impresionó a Irma* [...] *fue cuando algunos médicos cajetillas -que durante el peronismo eran fervientes peronistas y luego de la Libertadora pasaron a ser opositores acérrimos- ataron a sus autos los bustos de Perón y Evita, con el objetivo de sacarlos y arrastrarlos, mientras otros brindaban con champagne y cantaban* [...] *Como contraparte, los trabajadores de origen popular, sus compañeros, lloraban*”. (Héctor “Pelusa” Carrica, marzo 2001)

Estos acontecimientos, sumados a otros que vinieron luego, la llevaron a adoptar una actitud revisionista sobre la experiencia del peronismo.

Por su parte, la autoproclamada “Revolución Libertadora” -por intermedio del Decreto 4161- buscaba desperonizar el país. Bajo este prisma, la disputa se planteaba en términos de legitimidad, no de legalidad. En ese hiato, si bien Perón seguía siendo el productor de ideología por antonomasia, su exilio abrió un escenario de pluralización interpretativa y práctica sobre el significado del peronismo como experiencia histórica. Conforme evolucionó esta dinámica, se fueron configurando las distintas expresiones de la Izquierda Peronista, junto con el devenir situado de su ideología^21^.

Para graficar esta etapa, “Pelusa” narró las experiencias vividas junto a su madre, delineando así los caracteres que definieron el clima de época. Según sus palabras:

“…*había algunos grupos muy activos con los que ella toma contacto a través del gremio y se vincula a la Resistencia Peronista. Y te diría que crecí en ese clima* [...] *en las asambleas, acompañando a mi vieja* [...] *En los sindicatos militaban las distintas organizaciones juveniles peronistas* [...] *eran los que sostenían, junto con las agrupaciones políticas de los sindicatos, lo que era la vida del Peronismo* [...] *eran grupos muy decididos, de acción directa muchas veces*”. (Héctor “Pelusa” Carrica, marzo 2001)

Asimismo, en lo relativo a la enfermería, la eliminación del legado peronista también dejó su huella. La influencia de los organismos internacionales, sobre todo la Organización Panamericana de la Salud (OPS) y la Comisión Económica para América Latina y el Caribe (CEPAL), estimuló un perfil profesional vinculado con la división técnica del trabajo y la necesidad de capacitar un tipo de personal auxiliar especializado en servicios de baja complejidad. De tal suerte, las nuevas enfermeras debían incorporar conocimientos de gestión administrativa a sus tradicionales tareas técnico-científicas, aunque este grado de plasticidad no las alejó de la subordinación al poder médico. Por su parte, el desarrollo de escuelas universitarias tuvo un efecto paradojal porque, al mismo tiempo que jerarquizó la profesión, reforzó las asimetrías al interior del gremio de enfermería. Finalmente, desde un perfil verticalista y corporativo, las distintas asociaciones y federaciones del periodo se centraron en el desarrollo científico, educativo y laboral de la enfermería, desatendiendo las luchas sindicales, sociosanitarias y políticas^22^.

Con respecto a esto último, en 1958 se creó -bajo el amparo de los organismos internacionales antes aludidos- la Asociación Argentina de Enfermeras Diplomadas. La misma, una vez que obtuvo la personería jurídica, se dedicó principalmente a cuestiones relacionadas con la formación de enfermeras y no tanto a las problemáticas económico-corporativas de sus representadas. Bajo este prisma, participó del Censo de Enfermería, solicitado en 1956 por el Ministerio de Asistencia Social y Salud Pública de la Nación, con el objetivo de recibir los fondos para algunos programas internacionales de reforma^22^.

En tándem con lo anterior, en 1960 se creó la carrera de Enfermería de la Universidad de Buenos Aires, que funcionó en el marco del Hospital de Clínicas José de San Martín, hospital-escuela dependiente de la universidad. La carrera en cuestión vino a reemplazar a la Escuela de Enfermería creada por Carrillo, ahora con un perfil tecnocrático y escindido de la realidad social. En esa transición, la figura de Teresa Molina funcionó como un agente legitimador, desconociendo o modulando su compromiso con el pasado peronista^17^.

**Figura 1:**
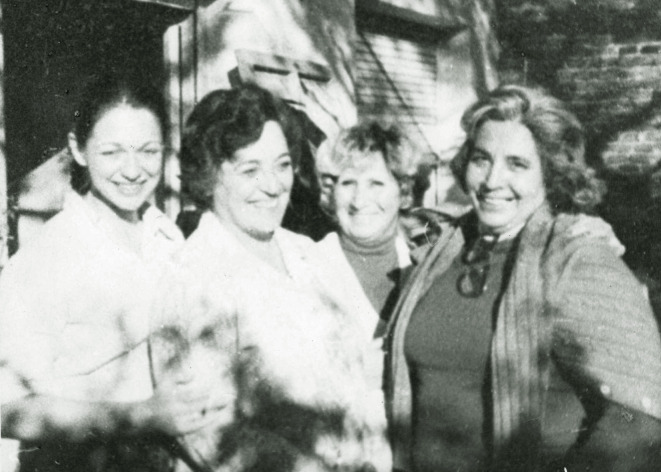
Integrantes del Curso de Auxiliares de Enfermería en la Escuela de Enfermería con sede en la Universidad de Buenos Aires. Circa 1961.

**Figura 2:**
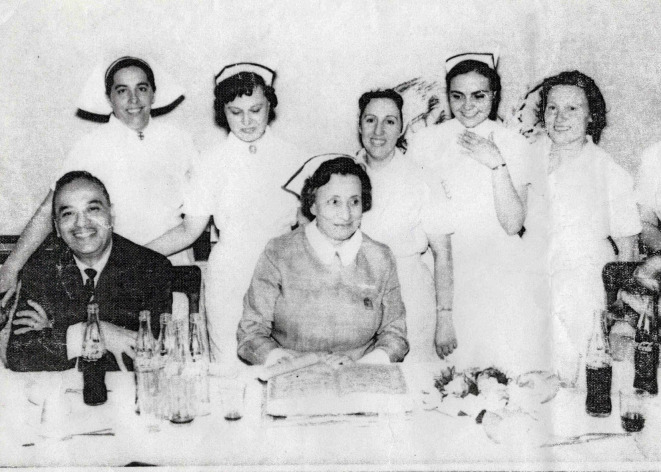
Integrantes del Curso de Auxiliares de Enfermería en la Escuela de Enfermería con sede en la Universidad de Buenos Aires. Circa 1961.

A lo largo de esta etapa, Carrica continuó desarrollándose profesionalmente. Primero obtuvo el título de Experta en Administración y Supervisión en Enfermería, otorgado por la Escuela Nacional de Salud Pública. Luego pasó a coordinar los Cursos de Auxiliares de Enfermería en la Escuela de Enfermería con sede en la Universidad de Buenos Aires (Figura 1 y Figura 2). Asimismo, llegó a ser la primera docente coordinadora de la Oficina Sanitaria Panamericana^19^.

Nota: En la foto se puede ver a Irma Carrica (primera persona a la derecha) y Teresa Molina, directora del curso (tercera persona de iquierda a derecha). Foto aportada por Graciela Ríos, compañera de Héctor “Pelusa” Carrica.

Nota: En la foto se puede ver a Irma Carrica (primera persona parada a la izquierda) y Teresa Molina, directora del curso (en el centro de la foto, sentada). Foto aportada por Graciela Ríos, compañera de Héctor “Pelusa” Carrica.

En ese recorrido, encontramos otro dato característico de su trayectoria: el hecho de habitar marcos institucionales desfavorables, capitalizando la legitimidad de estos para llevar adelante actividades coincidentes con su enfoque sobre la salud pública. En esta clave, por ejemplo, dictaba clases *ad-honorem* de formación que anticipaban algunas características del enfoque comunitario de las etapas subsiguientes. Según el testimonio de “Pelusa”:

“*Mi vieja restaba horas de sueño para dictar clases ad-honorem en los hospitales* [...] *Recuerdo los pasillos del Hospital Borda* [...] *Su objetivo en esas noches era capacitar a los empíricos* [...] *Sus clases eran más que originales, ya que los primeros en acudir eran los pacientes. De modo que, tanto las enfermeras como los pacientes participaban de esos talleres*”. (Héctor “Pelusa” Carrica, marzo 2001)

A partir de esta época, Carrica estuvo siempre junto a otras cinco compañeras que constituyeron su núcleo más directo de militancia y sociabilidad. Se trataba de las “viejas de la resistencia”, como las recuerda cariñosamente “Pelusa”. A través de las entrevistas, pudimos identificar que una de ellas era Rosa Haydée Cirullo de Carnaghi, también conocida como la “Tía Tota”.

### Consolidación del desarrollismo y cambio de estrategia (1958-1966)

Hacia 1958, Perón fue desarrollando, desde el exilio, una nueva estrategia para el movimiento. En lo electoral, puso a prueba su capacidad para incidir en los resultados y condicionar la evolución del régimen proscriptivo. En lo sindical, los nuevos liderazgos peronistas -sobre todo en los grandes sindicatos- acentuaron su rol defensivo y economicista, en desmedro de los objetivos políticos relacionados con el retorno de Perón. En consecuencia, muchos líderes gremiales -bajo el amparo de la Ley 14455- se volvieron más proclives a desarrollar una agenda propia, en diálogo con el régimen. De tal suerte, se abrió una nueva disputa dentro del peronismo: por un lado, los “duros”, que representaban la intransigencia frente al régimen y la confianza en la vía insurreccional; por el otro, los “blandos”, dispuestos a defender la “legalidad sindical” bajo una estructura de representación de carácter autocrático, centralizado y con altos niveles de burocratización. Frente a las dos alternativas extremas del movimiento, el General Perón adoptó un comportamiento pendular, tratando de capitalizar -en los distintos contextos- el peso de cada una de ellas para legitimar su conducción^23^.

En ese contexto, según el testimonio de “Pelusa”, Carrica formó parte del “sindicalismo combativo o de liberación”, que se oponía al “sindicalismo complaciente” expresado por Augusto Vandor. Bajo esa órbita sindical -siempre desde la Asociación Trabajadores del Estado- estableció vínculos con la línea de Amado Olmos, un importante dirigente del gremio de Sanidad que participó junto a “los duros” en distintas gestas nodales de la resistencia peronista.

Por otro lado, en 1965, Carrica participó -a pesar de la renuencia institucional a reconocerlo- en la fundación de la Federación Argentina de Enfermería, cuya presidencia quedó en manos de Teresa Molina^17^. En línea con los móviles de su precursora, la Asociación Argentina de Enfermeras Diplomadas, la Federación se creó bajo la égida de la Oficina Sanitaria Panamericana, que impulsó, por intermedio de Nydia Gordillo Gómez, el perfil que adoptaría la profesión luego de su incorporación a la educación superior^24^. Frente a la incógnita de por qué Irma participó de un espacio tan lejano a sus convicciones, asumimos que pudo tener que ver con su pertenencia institucional a la Escuela de Enfermería de la Universidad de Buenos Aires. Sin embargo, no descartamos que haya sido el resultado de una elección motivada por la voluntad de dar la lucha desde adentro y conocer la agenda que influiría en el devenir de su profesión.

### Las disputas por el sentido de “la comunidad” en el campo de la salud (1966-1971)

Tras el golpe de Estado de 1966 y luego de una fuerte devaluación, el programa económico del ministro Adalbert Krieger Vasena buscaba consolidar la posición de las industrias más “dinámicas” con financiación de capitales extranjeros, al tiempo que alimentaba el mercado especulativo. De tal suerte, vía un proceso de concentración económica y desnacionalización de empresas, se resintió la actividad de las pequeñas y medianas industrias ligadas al mercado interno, consideradas más “ineficientes”. A su vez, para estimular la inversión extranjera, el Estado congeló los salarios y aplicó criterios de racionalización de la producción que iban en desmedro de las condiciones laborales. Finalmente, con el objetivo de morigerar el déficit fiscal, aumentó las tarifas y redujo el empleo público^20^.

Para mayo de 1969, se produjo un hito en la historia de las luchas sociales en la Argentina: una importante manifestación de obreros y estudiantes sacudió la provincia de Córdoba, en lo que se conoció como el “Cordobazo”, que fue acompañado por levantamientos similares en otros lugares del país, constituyéndose en un fenómeno de alcance nacional. En ese contexto, las manifestaciones populares no solo expresaron los intereses económico-corporativos de la clase obrera, sino que pusieron en tela de juicio la legitimidad del conjunto de los aparatos represivos del Estado, sentenciando así el ocaso del régimen dictatorial. En esa gesta, la Confederación General del Trabajo de los Argentinos (CGTA) desempeñó un rol importante en la difusión de iniciativas antidictatoriales y en la articulación de distintos frentes de lucha, bajo la égida de dirigentes combativos como Raimundo Ongaro y Agustín Tosco^25^^,^^26^.

En el marco de esa central combativa, Carrica desarrolló una labor importante en torno a los derechos humanos, dentro de la Comisión de Solidaridad^17^. Junto a un grupo de mujeres que ella había conformado, se dedicaban a localizar a los presos políticos en las comisarías, a los efectos de evitar que los incomunicaran y los torturaran. A su vez, brindaban atención sanitaria y contención a los que, producto de la tortura, eran trasladados a hospitales y esposados a las camillas. Frente a esas situaciones, según Pelusa:

“*Iva Irma, con su uniforme, su cofia, y lo primero que hacía era sacar a los policías de al lado de la cama del paciente, para reconfortarlo, curarlo y transmitirle esperanza* [...] *También hacía mucho ‘despelote’, denunciando a los oficiales y jueces en la calle y ante la prensa, hasta que llegaran los abogados*”. (Héctor “Pelusa” Carrica, marzo 2001)

Estás formas de intervención se replicaron en distintos escenarios, pero el testimonio más elocuente que encontramos fue el de Luis Rodeiro. Él fue detenido luego del llamado “combate de William Morris”, estuvo preso y fue torturado en la Coordinación Federal de Buenos Aires. Según su relato, como producto de la tortura, tenía una quemadura pequeña pero profunda que estaba gravemente infectada. Frente a esa situación, la ayuda vino de quienes eran sus compañeros de celda y de la Comisión de Solidaridad^27^. Allí apareció la figura de Irma, a quien Luis recordó del siguiente modo:

“...con la humildad de las mujeres comprometidas, se convirtió en ala protectora de esa herida maloliente. Buscó los intersticios para curarla, protestó ante los jerarcas policiales, concertó citas con mis familiares para que coincidiéramos en la hora de visitas. Por cierto triunfó, y la herida se curó. Queda tan sólo una cicatriz que, en lo personal, más que el odio de los torturadores me recuerda siempre al amor solidario de Irma”.^27^

### Experiencias alternativas de participación social en salud

Como ya hemos señalado, entre 1966 y 1971 se manifestaron distintas experiencias alternativas de participación social en salud, que confrontaban con el espíritu de las iniciativas de “descentralización comunitaria” y encontraban sustento en los procesos de resistencia social y política contra la dictadura.

En un análisis preliminar, pudimos identificar algunas de las experiencias alternativas aludidas que, para el caso argentino, ponían en tela de juicio el sentido que la Ley 17102 le atribuía a la participación en salud. Por un lado, tenemos las intervenciones sanitarias en la Villa 31, en su origen motorizadas por el Consejo Mundial de Iglesias de 1968 y luego ligadas al Movimiento de Sacerdotes por el Tercer Mundo^28^. Por otra parte, en el marco de las luchas por higiene y salubridad en el trabajo, fueron relevantes los contactos entre el “sindicalismo combativo” y algunos profesionales de la salud comprometidos políticamente^29^. Finalmente, las “asambleas terapéuticas” del Hospital Melchor Romero de La Plata^30^ y las reflexiones político-pedagógicas derivadas de la intervención territorial del servicio de Salud Mental “El Lanús” que dependía del Hospital Interzonal de Agudos Evita de Lanús, cuyos profesionales realizaban tareas de articulación y organización territoriales^31^^,^^32^, también constituyeron experiencias claves en esa disputa.

Durante esta etapa, Carrica continuaba coordinando los cursos de Auxiliares de Enfermería en la Escuela de la Universidad de Buenos Aires. Según el testimonio de María Lucrecia “Maluca” Ciriani (enfermera militante de la izquierda peronista en salud), si bien Carrica no contaba con el título de grado para dar clases en la carrera universitaria, era parte de su concepción trabajar junto a estudiantes de origen humilde como los que aspiraban a ser auxiliares. Por el contrario, el resto de las docentes “*miraban con desprecio esa labor*”. Asimismo, sabían que “*Irma era peronista y defendía las causas populares*”, lo que reforzaba aún más el distanciamiento con sus pares.

Al recordar el trato con los estudiantes, “Maluca” sostuvo que era notoria la pertenencia de Carrica a los sectores populares, dada la facilidad que tenía para interpelarlos con su discurso e interactuar con sus ideas. En ese sentido, afirmó:

“*Si yo la tuviese que caracterizar, te diría que era una mujer sencilla, del pueblo, campechana* […] *Viste esa gente que fácilmente entiende a las personas, que hace las cosas sencillas* […] *muy querible, simpática, siempre sonriente* […] *Tenía un gran compromiso con lo popular, no era sectaria y tenía una firme preocupación por garantizar el acceso a la salud para todos*”. (María Lucrecia “Maluca” Ciriani, marzo 2023)

A su vez, la entrevistada destacó las diferencias entre el enfoque que le imprimía Carrica a sus cursos y el del resto de las instancias de formación de la Escuela de Enfermería de la Universidad de Buenos Aires. En ese sentido, sugirió que:

“*En aquel entonces* [1971] *existía una formación muy tecnocrática, centrada en el uso de aparatos complejos y cuestiones formales, muy alejada de las necesidades de la gente* […] *Nadie te enseñaba nada sobre salud pública, ni sobre epidemiología. No se hacía ninguna referencia a Carrillo, ni tampoco al surgimiento del Ministerio de Salud* […] *Irma era la única entre las docentes que acordaba con quienes discrepábamos con ese tipo de formación y militábamos para cambiarla* […] *Yo creo que sus alumnas tenían mejor formación que nosotras, te digo. Menos técnica, pero más social, más popular*”. (María Lucrecia “Maluca” Ciriani, marzo 2023)

En consonancia con esto, pudimos saber -a través del testimonio de “Pelusa”- que Irma se había ganado el apodo de “la enfermera de los pobres”, dada su insistencia en trasladar a sus estudiantes hacia los barrios populares y ponerlos en contacto con las demandas y los procesos organizativos de los excluidos. Bajo este prisma, realizaban “*...actividades de educación y prevención, APS y a menudo levantaban centros comunitarios en distintos barrios populares, con la participación activa de los vecinos y vecinas*” (Héctor “Pelusa” Carrica, marzo 2001).

### Del Consejo Tecnológico a la gestión de la Universidad Nacional y Popular de Buenos Aires (1971-1974)

En 1971, fruto del desgaste del régimen, se abrió un escenario que convalidaba la posibilidad de una salida electoral, en el marco del Gran Acuerdo Nacional (GAN) impulsado por las negociaciones entre el presidente de facto Alejandro A. Lanusse y Perón.

Poco antes, en 1970, desde la oposición se había conformado una alianza pluripartidaria y multisectorial llamada “La Hora del Pueblo”. Esta alianza fue una de las principales interlocutoras que tuvo el régimen para encaminar una “salida negociada” hacia la apertura electoral. Sin embargo, el paradestinatario de estas interlocuciones era Perón, a quien se le reclamaban algunas concesiones desde el régimen: por un lado, que condenara el accionar de la guerrilla; por el otro, que los militares continuaran teniendo protagonismo en el próximo elenco de gobierno^33^.

En ese contexto, los debates nacionales sobre el sistema de salud atravesaban un proceso de transición que iba de la hegemonía del “seguro social” a la del “servicio universal” y, conforme se vislumbraba la posibilidad del retorno de Perón, ganaba terreno este último paradigma^34^.

El único estudio que se ocupó de investigar pormenorizadamente los proyectos de salud de los equipos reclutados por el peronismo fue el de Susana Belmartino y Carlos Bloch^34^. Dicho estudio tuvo el mérito de realizar esa reconstrucción, enmarcándola en los debates que le darían forma al proyecto de ley del Sistema Nacional Integrado de Salud de 1974, muy significativo para la izquierda peronista en salud. No obstante, desde un enfoque neo-institucionalista^2^, los autores separan el plano institucional de lo que acontece en la sociedad civil, ubicando las transformaciones sociales significativas solo en esta última esfera. Bajo este prisma, no ven diferencias sustantivas entre los proyectos de salud de los tres equipos político-técnicos más importantes del peronismo de aquel entonces^34^.

Por otra parte, existen interesantes reconstrucciones generales^11^^,^^33^^,^^35^^,^^36^ sobre el funcionamiento y los objetivos de los equipos aludidos que, aunque nos aportan datos fundamentales sobre la izquierda peronista en salud, no se ocupan específicamente de ella. Con base en esos trabajos, identificamos que, a partir de 1971, la izquierda peronista en salud comenzó a elaborar una teoría más sistemática en torno a las experiencias de la etapa anterior. Sin pretensiones de exhaustividad y aunque -como señala Friedemann^11^ las redes de pertenencia pueden ser múltiples y no necesariamente excluyentes, al menos dos grupos contribuyeron a darle forma, no sin discrepancias, al incipiente proyecto sanitario de la izquierda peronista en salud. Por un lado, el grupo ligado a la figura de Rolando García, que luego integró el Consejo Tecnológico, con la participación en el área de salud de figuras como Floreal Ferrara, José Carlos Escudero y Mario Hamilton. Por el otro, los equipos político-técnicos de la Juventud Peronista, con Felipe Aguerre y Sergio Laplume en salud, quienes contaron con la asesoría de Mario Testa. Por aquellos años, también, un espacio de debate fundamental para la izquierda peronista en salud fue la revista *Ciencia Nueva*, en la que se fueron cristalizando sus principales preocupaciones en torno a dos ejes: la relación entre ciencia e ideología y el vínculo entre identidad y modernización^12^. Estos debates fueron sintetizados en el segundo número de la revista *Bases para un Programa Peronista de acción de gobierno: Salud*.

Hacia finales de 1972, según Ernesto Villanueva -por entonces referente de los equipos político-técnicos- quienes habían hecho un trabajo más sistemático para confeccionar un “programa de gobierno” eran los integrantes del Consejo Tecnológico, cuyas reflexiones eran preponderantes dada la trayectoria profesional y el prestigio de algunos de sus integrantes.

En virtud de nuestra pesquisa, el testimonio que Mario Hamilton brinda en su autobiografía resulta muy relevante, no solo por sus condiciones técnicas y su participación en el Consejo Tecnológico, sino por las redes políticas y profesionales que fue construyendo desde fines de los años sesenta. Por un lado, en el marco de la Encuesta Nacional de Salud, donde coordinó un equipo en el que participaron importantes cuadros técnicos de la izquierda peronista en salud; posteriormente, en un programa internacional de investigación en salud, en el que trabajó junto a otra figura clave como Mario Testa; y, finalmente, en la Escuela de Salud dependiente de la Universidad de Buenos Aires, donde estableció vínculos con José Carlos Escudero y Osores Soler^37^.

En cualquier caso, revisando el segundo fascículo de la revista *Bases para un Programa Peronista de acción de gobierno*, pudimos reconstruir algunas de las ideas nodales de la izquierda peronista en salud, aunque resulta importante -como ya hemos sugerido- la posibilidad de abogar por una pluralización del objeto, rastreando los matices y discrepancias entre sectores del ala izquierda del sanitarismo peronista.

Por un lado, en consonancia con las experiencias de la etapa anterior (1966-1971), los profesionales debían despojarse de sus símbolos corporativos y de asimetría en términos de saber-poder, para adentrarse de manera paritaria en el abordaje colectivo del “proceso de salud- enfermedad-atención”^38^. Tal como lo indicaba el número de salud de la revista:

“Los trabajadores de salud en equipo interdisciplinario, deben integrar y orientar el proceso siempre en contacto con el sistema productivo y creativo en un trabajo de participación, ensayo y error, con desmitificación del rol científico […] donde los roles sociales se vuelven fluidos e intercambiables en la relación médico-paciente, sano-enfermo, profesor-alumno, jefe-subordinado”.^38^

Según la bibliografía especializada^17^ y los testimonios, Carrica formó parte del Consejo Tecnológico dirigido por Rolando García y participó en la confección de los proyectos de salud presentados por el Frente Justicialista de Liberación Nacional en las elecciones de 1973.

### Postas de salud, equipos de solidaridad y lazos intergeneracionales de militancia

En 1972, Carrica continuaba al frente de los cursos de Auxiliares de Enfermería. Ya para ese entonces, “Pelusa” había ingresado a trabajar en el Hospital Rivadavia, en el área de mantenimiento, a través de los contactos que tenía su madre. Como delegado de la Asociación de Trabajadores del Estado en el hospital, formó parte de la Juventud de Trabajadores Peronistas, que constituía la pata sindical de los “Frentes de Masas” de la Tendencia Revolucionaria, cuyo objetivo era disputar espacios y lógicas de construcción sindical con los sectores que formaban parte de la llamada “burocracia”^39^.

Para ese entonces, “Maluca” cursaba la Licenciatura en Enfermería de la Universidad de Buenos Aires y participaba en el centro de estudiantes. Desde ese espacio, aparte de las ya citadas desavenencias con el perfil de formación, bregaban por ampliar la disponibilidad horaria para estudiantes que quisieran trabajar. Para canalizar ese reclamo, tomaron el establecimiento. Según el relato de la entrevistada:

“*Debe haber sido en 1972, porque esa escuela te exigía estudiar desde las ocho de la mañana hasta las cinco de la tarde* […] *entonces nadie podía trabajar, era un espacio que apuntaba a la élite* […] *Por eso tomamos el establecimiento* […] *Fue muy interesante* […] *encerramos a todas las docentes en un aula, nos metimos donde estaba la directora y le dijimos: ‘o haces esto o renuncias o te tiramos por la ventana’* […] *Fue un poco brutal. Imaginate, todas las docentes gritando… Irma se sonreía como aprobándonos*”. (María Lucrecia “Maluca” Ciriani, marzo 2023)

Fue entonces que, luego de hablar con el decano, lograron imponer a la licenciada Marta Rojas como directora. No pudieron poner a Carrica porque no contaba con el título universitario, pero entablaron un vínculo muy cercano con ella. De hecho, para el año 1972, Irma le ofreció a “Maluca” que entrara a trabajar como “enfermera nochera” en el Hospital Rivadavia. Allí conoció a “Pelusa”, con quien salió por algún tiempo. En el marco del hospital, si bien pertenecían a espacios políticos distintos, convergieron en las actividades sindicales. Según su testimonio:

“Por esa época se dio la toma del hospital, antes de la asunción de Héctor Cámpora en el 73. Fueron muchos días los que vivimos en el Rivadavia, nos quedábamos día y noche para garantizar que las nuevas autoridades fueran compañeros. En la cocina del hospital había una compañera, muy querida, muy peronista, llamada Tota. Una mujer grande, conocida por todos en el hospital [...] Muchas de las reuniones se hacían en su cocina y durante el tiempo que duró el conflicto ella garantizaba la comida de todos”.^27^

En este fragmento volvemos a ver la importancia que tenían los lazos intergeneracionales con las llamadas “tías” o “viejas de la resistencia”, así como el hecho de que la toma de establecimientos era un mecanismo de lucha extendido entre las juventudes revolucionarias de aquel entonces. Luego de la asunción de Cámpora, la Juventud de Trabajadores Peronistas desarrolló en el hospital una serie de jornadas voluntarias de reparación y actividades participativas abiertas a la comunidad. Tanto “Pelusa” como “Maluca” recordaron esas jornadas como una forma alternativa de habitar el espacio público e interactuar con los usuarios.

Por último, en el marco de la entrevista con “Maluca” Ciriani, pudimos acceder a una fuente primaria titulada “*Puestos de control sanitario y de orientación*”^40^. El documento en cuestión reseñaba las características de los puestos de control “fijo” y “móvil” que acompañarían la movilización a Ezeiza, en ocasión del retorno de Perón al país. Según la entrevistada, el documento le fue entregado por la propia Carrica, quien era responsable de parte de la organización de este dispositivo sanitario.

### La gestión de la Universidad Nacional y Popular de Buenos Aires en manos de la izquierda peronista

Hacia 1973, el proyecto de la izquierda peronista en salud comenzó a consolidar cierta carnadura institucional, cimentada en sus etapas anteriores. Por un lado, con la asunción de Mario Testa como decano de Medicina, en el marco de la Universidad Nacional y Popular de Buenos Aires que tenía como rector a Rodolfo Puiggrós^13^^,^^41^^,^^42^. Por el otro, con Floreal Ferrara al frente del Ministerio de Salud y Acción Social de la provincia de Buenos Aires, durante el Gobierno de Bidegain^14^^,^^43^^,^^44^^,^^45^.

Dentro de la Universidad Nacional y Popular de Buenos Aires, en el marco del periodo que Friedemann^13^ ha llamado “institucionalización inconclusa” (29/05/1973 - 17/09/1974), proliferaron una serie de institutos de investigación y proyectos de extensión que -bajo un influjo significativo de la pedagogía de Paulo Freire- aspiraban a construir una “universidad al servicio del pueblo”. En el caso de la Facultad de Medicina, se destacó el Instituto de Medicina del Trabajo. En el caso de la Facultad de Farmacia y Bioquímica, junto con la de Medicina, el influjo de la izquierda peronista en salud se vio reflejado en el Instituto del Medicamento. El mismo fue creado bajo el decanato de Marcelino Cereijido^13^^,^^29^.

En los albores de esta experiencia de gestión, la Juventud Universitaria Peronista asumió un protagonismo inusitado en la designación de autoridades y en el propio gobierno universitario^13^. Según el testimonio de Marcela Durrieu, por entonces referente de esa agrupación en la Facultad de Medicina, sus integrantes se presentaban como “comisarios políticos” frente al decano que ellos mismos habían designado.

Sin embargo, esta situación se modificó drásticamente tras la renuncia del primer decano, el Dr. Tomás A. Mascitti. Fue entonces que Ernesto Villanueva, quien estaba a cargo de la Secretaría General del Rectorado, les acercó la propuesta de Mario Testa, un sanitarista que tenía experiencia internacional en planificación de políticas de salud y había formado parte del grupo de creadores del método CENDES/OPS^46^. No obstante, para 1971, ya había reflexionado sobre las consecuencias negativas que acarreaba dicho método, razón por la cual había retornado a la Argentina^47^. A través de Sylvia Bermann y Arnaldo Torrents, llegó a participar -como ya dijimos- en los equipos político-técnicos de la Juventud Peronista. En 1973, Puiggrós lo contactó para que asuma como decano^13^. Hasta ese momento, según el relato de Durrieu, ella estaba como secretaria privada, haciendo las veces de vicedecana. Esta situación se modificó con la llegada de Testa. Según el relato de Durrieu:

“...*Mario hizo cambios interesantes. Nombró, como primera medida, un secretario académico que era un tipo muy serio. Reordenó la cuestión académica y volvió a convocar docentes, haciendo que la facultad volviese a funcionar como tal* [...] *Introdujo cambios pedagógicos importantes: desde qué se enseñaba hasta la posibilidad de tener un régimen de vestimenta más laxo, pasando por la habilitación de espacios para realizar asambleas. De hecho, él mismo realizaba una suerte de asambleas para poner en común las políticas universitarias con los estudiantes* [...] *También planteaba la cuestión social de la medicina, el vínculo de la formación universitaria con las problemáticas sociales*”. (Marcela “Hormiga” Durrieu, marzo 2023)

En el marco de los cambios impulsados por Testa, uno de los ejes principales tenía que ver con la reforma del plan de estudios, que transformaría la Facultad de Medicina en una Escuela de Ciencias de la Salud, incorporando al currículo el concepto de niveles progresivos de complejidad en la formación. En ese sentido, el tronco común para las carreras de Enfermería, Nutrición y Medicina incluía cuatro áreas correspondientes al primer nivel de complejidad, a saber: social, morfológica, fisiológica y patológica^37^.

Asimismo, en tándem con la idea de vincular la actividad universitaria con la resolución de las problemáticas sociales y políticas del país, la gestión de la izquierda peronista en salud intentó articular la formación, la investigación y la extensión con los procesos organizativos y reivindicativos de los sectores populares. En muchos casos, esa articulación se dio a través de los “Frentes de Masas”. En ese sentido, si bien existen numerosos trabajos sobre dichos “Frentes”, la mayoría de ellos se centra en la perspectiva de las “organizaciones de superficie”^39^^,^^48^^,^^49^^,^^50^ y no en el rol de las redes profesionales e intelectuales, con las notables excepciones del trabajo de Friedemann^51^ sobre los Centros Piloto de Investigación Aplicada, entre otros proyectos de la Universidad Nacional y Popular de Buenos Aires, y el de Spinelli y Martin^29^ sobre el Instituto de Medicina del Trabajo.

De acuerdo con la investigación de Spinelli y Martin, el Instituto de Medicina del Trabajo buscaba articular los procesos técnico-científicos con las demandas de los trabajadores organizados, postulando la necesidad de un rol protagónico de estos últimos en el control de la higiene y la seguridad laborales. En ese camino, conforme con las preocupaciones de la izquierda peronista en salud en las etapas anteriores, planteó:

“...una tensión permanente con el rol adjudicado a los médicos y a las ciencias de la salud en general. A través del lugar destinado a los obreros en el control de su salud, puso en cuestión el criterio de medicina del trabajo vigente, el rol de los profesionales, de la universidad y de la ciencia en su conjunto”.^29^

A su vez, tales modificaciones en la práctica y el pensamiento sanitarios intentaban incidir en los debates sobre la estrategia política y el rol de los intelectuales. En ese sentido, al hablar sobre la influencia de la interpretación gramsciana del grupo Pasado y Presente en el Instituto de Medicina del Trabajo, Ricardo Saiegh, quien fuera su director, señaló:

“En el contexto de un cierto debate que había en América Latina, entre el foquismo y lo que podríamos llamar el insurreccionalismo […] no compartíamos totalmente el criterio foquista y seguíamos pensando que si iba a haber algún cambio histórico tenía que surgir del pueblo […] [De modo que] la relación del instituto con las organizaciones obreras ya era un germen de algo nuevo”.^52^

En el mismo sentido, aunque recuperando otra faceta de la acepción gramsciana de “espontaneidad” en Pasado y Presente^53^, Rubén Efron, compañero de Saiegh en el Instituto de Medicina del Trabajo, detalló los fundamentos principales de la crítica que ellos le hacían a las posiciones vanguardistas de aquel entonces. En el marco de esa reconstrucción, señalaba lo siguiente:

“Nosotros imaginábamos que la participación en las comisiones obreras tenía que ser universal […] En términos ideales, no debía haber ningún trabajador que no participara […] Sin embargo, la intención no era imponerlo como algo obligatorio, sino que buscábamos que la participación en los asuntos comunes se fuese naturalizando […] criticábamos la concepción vanguardista o militarista […] [y esa crítica] se apoyaba en la idea de que el saber tenía que ser producto de una apropiación colectiva […] Digamos que, al aceptar que el saber era colectivo, nos ubicábamos en un lugar distinto al de las vanguardias”.^54^

Partiendo de estas interpretaciones, los jóvenes del Instituto de Medicina del Trabajo buscaban abonar un “proceso de reinscripción” dentro de la historia del Movimiento Justicialista, a partir de un diálogo intergeneracional con sectores de la “ortodoxia”. En ese sentido, no dejaban de reconocer la relevancia del período justicialista y de Ramón Carrillo como una de sus figuras señeras en el campo de la salud^29^, pero lo hacían mixturando esos contenidos con sus propias trayectorias políticas.

En esta clave, allende las conocidas desavenencias entre los representantes del grupo Pasado y Presente y parte de la izquierda peronista^55^^,^^56^^,^^57^, las reflexiones de ambos grupos -al menos en este caso- estimularon intervenciones y proyectos políticos afines.

No obstante, Ernesto Villanueva se sorprendió al saber del influjo gramsciano en el marco del Instituto de Medicina del Trabajo, dado que el teórico sardo abogaba por una “*lucha de índole cultural*” y “*descreía de la revolución en términos de ‘toma del poder*’”. Como contraparte, propuso otra serie de teóricos que sí eran relevantes -según él- para la Tendencia Revolucionaria, principalmente desde la experiencia de las cátedras nacionales. Sin embargo, expuso un argumento sobre por qué podían darse esas mixturas:

“*Una cosa eran las actividades de gestión y otra era la forma que adoptaban los programas desde los saberes disciplinares. Mientras las distintas facultades respetaran lo que era nuestro ‘ADN’, el vínculo de la universidad con las problemáticas sociales y políticas del país, se aceptaban las distintas adaptaciones disciplinares*”. (Ernesto Villanueva, abril 2023)

En cualquier caso, Irma Carrica desempeñó un rol significativo, tanto en el Instituto de Medicina del Trabajo como en el proyecto general de la Facultad de Medicina. Según Testa^58^, Carrica formó parte del “grupo de coordinación de la facultad” que, en un plano de igualdad, abordaba las problemáticas gremiales, académicas y organizativas, planteadas tanto desde la gestión como desde el estudiantado. Asimismo, para Rubén Efron, fue una de las personas más comprometidas con las “Jornadas Nacionales de Medicina del Trabajo al Servicio de los Trabajadores”, organizadas por el instituto en noviembre de 1973. Según su testimonio:

“...ella tuvo una participación activísima con los trabajadores de Ford, para terminar con las intoxicaciones producidas por el plomo [...] Se llegó a realizar una asamblea que -según recuerdan los compañeros- fue de las más grandes de la historia de la fábrica, en la que participaron como 700 trabajadores. La misma tendía a organizar la forma de implementar el trabajo de investigación [...] Irma fue el corazón de ese proyecto que llegó a cuestionar el poder de la gobernanza sindical burocrática y motivó el nerviosismo de ciertos funcionarios”.^59^

**Figura 3:**
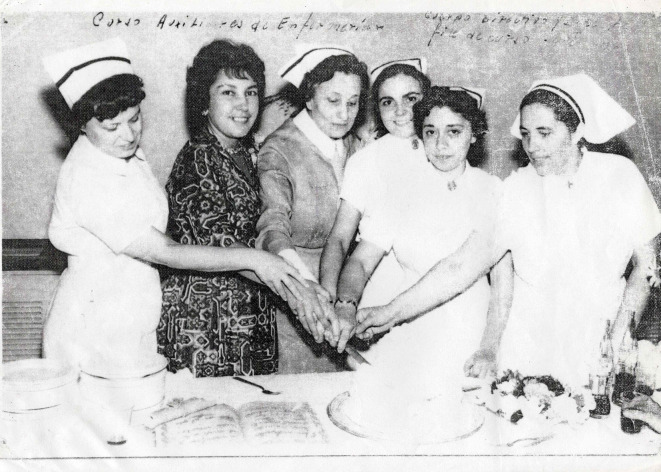
Irma Carrica junto a colegas.

En esta clave, tanto Testa como Efron recordaron a Irma como una profesional y militante muy comprometida, que no disociaba su palabra y sus ideas de su práctica cotidiana (Figura 3). La retrataron como una mujer apasionada, lúcida e inteligente, que tenía mucha facilidad para interactuar con las demandas y procesos organizativos del pueblo^58^^,^^59^.

Nota: Irma Carrica es la primera persona a la izquierda. Foto aportada por Graciela Ríos, compañera de Héctor “Pelusa” Carrica.

Asimismo, desde la Unidad Básica Revolucionaria que funcionaba en el Hospital de Clínicas José de San Martín; y luego en el marco de la Dirección del mismo hospital^19^, Irma Carrica impulsó la articulación de instancias universitarias con actividades territoriales del Movimiento Villero Peronista, en coordinación con la Juventud de Trabajadores Peronistas y la Juventud Universitaria Peronista^27^. Desde esos espacios, por ejemplo, se realizaban intervenciones sanitarias en la Villa de Retiro, junto con el Padre Mugica. Según el relato de “Pelusa”:

“*Ahí generamos el nacimiento del MVP* [Movimiento Villero Peronista], *que era una de las estructuras de nuestra organización. Y montamos innumerables centros de trabajo sanitario en el territorio. Todo eso lo íbamos haciendo medio con el trabajo que hacía Irma* [...] *Ella hacía la base de enfermería, llevaba a los estudiantes, a los futuros médicos. Y nosotros laburábamos el tema territorial con los vecinos de las villas* [...] *desde mucho tiempo, fuimos, además, acompañando todo ese proceso que hubo en la universidad y en la salud, donde había innumerable cantidad de médicos progresistas* [...] *que participaban en las famosas ‘Cátedras de Medicina para el Trabajo*’”. (Héctor “Pelusa” Carrica, octubre 2018)

No obstante, conforme avanzaban los enfrentamientos de la izquierda peronista con Perón y con sectores de la derecha peronista, los espacios para realizar esta clase de actividades se fueron acotando hasta desaparecer. Para septiembre de 1973, conforme escalaban los antagonismos, fue asesinado el secretario general de la Confederación General del Trabajo, José Ignacio Rucci. A su vez, en el marco de una embestida represiva contra la izquierda peronista, Puiggrós fue desplazado del Rectorado en octubre. Sin embargo, fruto de la presión de la Juventud Universitaria Peronista y otros aliados, evitaron que asuma Alberto Banfi, por entonces decano de Odontología, y lograron imponer a Ernesto Villanueva^11^. Para ese entonces, renunciaba a su cargo Mario Testa. En su lugar, asumió Ricardo Saiegh^13^.

Para marzo de 1974, luego de que se aprobara la “Ley Taiana” para la universidad, asumió como rector normalizador el “conservador popular” Vicente Solano Lima, quien hasta ese momento ocupaba el cargo de secretario general de la Presidencia de la Nación. Tras el fallecimiento de Perón, Solano Lima renunció y por un breve lapso asumió Raúl Laguzzi. No obstante, cuando Isabel Martínez de Perón accedió a la Presidencia, desplazó a Jorge Taina del Ministerio de Educación y lo reemplazó por Oscar Ivanissevich. Este, desde un nacionalismo católico de extrema derecha, emprendió una “cruzada contra el marxismo”. Bajo este prisma, nombró como rector de la Universidad de Buenos Aires al autoproclamado fascista Alberto Ottalagano, quien desarticuló todas las iniciativas impulsadas durante el proceso de “institucionalización inconclusa” del proyecto universitario de la izquierda peronista, al tiempo que persiguió políticamente a sus referentes y los desplazó de sus cargos^11^.

Conforme aumentaba la represión estatal y paraestatal, los grupos armados tendieron a replegarse, dejando desguarnecidas a sus “organizaciones de superficie”^60^. En distintas entrevistas, “Pelusa” contó que, luego del golpe de Estado de 1976, intentó convencer a su madre de que se exilie con él, a lo que ella respondió que se quedaría luchando por el esclarecimiento de la situación de los desaparecidos. Fue en una de esas conversaciones que, frente a la insistencia de su hijo, ella evocó la frase de Evita: “el que no da todo, no da nada”, para quedarse y continuar con la búsqueda. Este encuentro tuvo lugar dos meses antes de la detención y posterior desaparición de Irma Carrica^18^.

Dada su exposición pública, Carrica le había pedido a su hermano que se quedara con ella en su casa del barrio porteño de Liniers. Fue allí donde, el 18 de abril de 1977, irrumpió un grupo de tareas y los secuestró a ambos. Como sobreviviente, el hermano de Irma dio testimonio de que ella fue trasladada al I Cuerpo de Ejército. Irma continúa desaparecida hasta el día de hoy.

## PALABRAS FINALES

A lo largo de nuestro recorrido, hemos abordado distintos aspectos del proyecto sanitario de la izquierda peronista en salud, a través de la trayectoria política, sindical y profesional de una de sus referentes. En lo concerniente a la disputa por el sentido de “la comunidad” en el campo de la salud, observamos que la originalidad de la izquierda peronista en salud tuvo que ver con la adopción de principios epistemológicos alternativos y el desplazamiento hacia contextos epistémicos situados en la periferia, tomando a la participación social como un pilar heurístico para la producción de tecnologías y conocimientos técnicos asociados a las necesidades, saberes y prácticas de las clases populares. En esa revisión de la episteme moderna, la izquierda peronista en salud puso en tela de juicio los principios propalados por el llamado “modelo médico hegemónico”^61^, junto con las lógicas autoritarias que configuran el “sentido práctico” del campo y el *habitus* médicos.

Asimismo, detectamos que la izquierda peronista en salud ensayó un intento de reinscripción en la historia del peronismo. En ese camino, se enfrentó con una serie de “significantes operantes”^62^, propios de las tramas discursivas y culturales con las que coexistió e interactuó, que modularon el alcance de sus propias prácticas y paradigmas alternativos, encorsetando su imaginación institucional y política. Sin embargo, tales significantes no pudieron cauterizar definitivamente la plasticidad de lo político, por lo que la izquierda peronista en salud, como el resto de los actores intervinientes, retuvo márgenes de agenciamiento y un devenir político-ideológico propios. Por otro lado, valiéndonos de la variación de escalas^63^, descubrimos diferentes trayectorias político-ideológicas dentro de la izquierda peronista en salud. Bajo este prisma, abordamos algunos de los matices y diferencias que existieron en el interior de la izquierda peronista, así como con otros sectores del movimiento identificados con la “ortodoxia” o con la “burocracia”.

En todos los casos, constatamos que el campo de la salud no fue ajeno a los procesos políticos, sociales y económicos de su tiempo, sino que tanto sus discusiones como sus prácticas estuvieron fuertemente atravesadas por los acontecimientos que definieron el contexto nacional e internacional de los distintos periodos. Dentro de ese campo, Carrica se valió de los capitales simbólicos, culturales y sociales de los que disponía, a los efectos de disputar las lógicas que regían los marcos institucionales y políticos que habitó. Desde esa perspectiva, trató de ensanchar el horizonte de lo posible, a los efectos de hacerlo converger con su lectura estratégica del porvenir. No lo hizo en soledad, sino como parte de un actor histórico colectivo que hemos identificado como izquierda peronista, sin desconocer su heterogeneidad, sus conflictos internos y sus límites históricos.

En el marco de esa trayectoria, Carrica llevó adelante funciones intelectuales, pero lo hizo conforme con el estilo del “intelectual orgánico”, es decir, no “en el núcleo intrínseco de las actividades intelectuales [sino] en el conjunto del sistema de relaciones en el cual dichas actividades (y, por lo tanto, los grupos que las personifican) se encuentran en el complejo general de las relaciones sociales”^64^. 

En ese sentido, buscó articular la concepción del mundo que tenían los “sencillos” con una teoría general sobre el funcionamiento de la sociedad y su posible transformación, a los efectos de disputar críticamente el “sentido común” hegemónico. Por lo tanto, no se ciñó a una lógica “particularista” o “económico-corporativa”, sino que buscó anudar, a través de la política, el campo intelectual con la práctica cotidiana de las clases subalternas. En esta clave, funcionó como un nexo de interlocución y “traducción”^65^ entre la cosmovisión práctica de los sectores populares y la abstracción teórica de las franjas medias intelectuales, con el fin de construir un proyecto de hegemonía alternativa que tuviese bases materiales concretas^64^.

Esto no invalida que, como otros sectores de la izquierda peronista, pueda haber incurrido, desde sus núcleos de sociabilidad política e intelectual, en lógicas de reificación del pueblo o de la clase obrera, propias del romanticismo o de ciertas variantes de la teoría marxista respectivamente. En lo concerniente al primer caso, el pueblo podía asumir, en el plano discursivo, una consistencia ontológica de carácter cultural. Bajo este prisma, obraba siempre de forma homogénea y transparente, revelando -en su folklore y en su historia- fragmentos de una verdad insoslayablemente ligada al espíritu de la Nación^66^. Esta concepción, a su vez, maridaba adecuadamente con ciertas modulaciones discursivas de los antagonismos planteados por el peronismo: entre “imperialismo” y “Nación”, entre “oligarquía” y “pueblo”, etc.^21^^,^^67^. Asimismo, en lo relativo al influjo del marxismo, la clase obrera podía adoptar -en términos narrativos- un carácter sustantivo, a los efectos de convalidar la existencia de un correlato necesario entre la base económica y la forma “real” de la conciencia. Ambas operaciones teóricas alimentaban un doble movimiento discursivo y práctico: por un lado, tendían a esencializar el comportamiento y la conciencia del supuesto actor protagónico del proceso revolucionario, para luego -desde una lógica vanguardista- suplantarlo cuando no actuaba según lo esperado^60^.
